# Manure application increased crop yields by promoting nitrogen use efficiency in the soils of 40-year soybean-maize rotation

**DOI:** 10.1038/s41598-020-71932-9

**Published:** 2020-09-10

**Authors:** Wei Hua, Peiyu Luo, Ning An, Fangfang Cai, Shiyu Zhang, Kun Chen, Jinfeng Yang, Xiaori Han

**Affiliations:** College of Land and Environment, Shenyang Agricultural University, Shenyang, 110866 China

**Keywords:** Plant sciences, Environmental sciences

## Abstract

It is great of importance to better understand the effects of the long-term fertilization on crop yields, soil properties and nitrogen (N) use efficiency in a rotation cropping cultivation system under the conditions of frequent soil disturbance. Therefore, a long-term field experiment of 40 years under soybean-maize rotation was performed in a brown soil to investigate the effects of inorganic and organic fertilizers on crop yields, soil properties and nitrogen use efficiency. Equal amounts of ^15^N-labelled urea with 20.8% of atom were used and uniformly applied into the micro-plots of the treatments with N, NPK, M_1_NPK, M_2_NPK before soybean sowing, respectively. Analyses showed that a total of 18.3–32.5% of applied N fertilizer was taken up by crops in the first soybean growing season, and that the application of manure combining with chemical fertilizer M_2_NPK demonstrated the highest rate of ^15^N recovery and increased soil organic matter (SOM) and Olsen phosphorus (Olsen P), thereby sustaining a higher crop yield and alleviating soil acidification. Data also showed that no significant difference was observed in the ^15^N recovery from residue N in the second maize season plant despite of showing a lower ^15^N recovery compared with the first soybean season. The recovery rates of ^15^N in soils were ranged from 38.2 to 49.7% by the end of the second cropping season, and the residuals of ^15^N distribution in soil layers revealed significant differences. The M_2_NPK treatment demonstrated the highest residual amounts of ^15^N, and a total of 50% residual ^15^N were distributed in a soil layer of 0–20 cm. Our results showed that long-term application of organic fertilizers could effectively promote N use efficiency by increasing SOM and improving soil fertility, and thus leading to an increase in crop yields. This study will provide a scientific reference and guidance for improving soil sustainable productivity by manure application.

## Introduction

Nitrogen (N) is an essential element for crop growth and development. N fertilizer plays a vital role in ensuring the steady growth of crops and satisfying the requirements of an increasing population^1–5^. Therefore, high-input of chemical N fertilizer is globally considered to be one of most important practices to improve crop yield^6^. Especially, the amounts of nitrogen fertilizer used in China reached 23,814,000 tons in 2011, and account for 35% of the total amount of the world's nitrogen fertilizer application. As the largest producer and consumer of nitrogen fertilizer, N fertilizer has been overused by Chinese farmers because of limited understanding on the relationship between N input and grain yield, but the growth rates of crop yields still are much lower than the expected crop yield growth generated through the application of N fertilizer, resulting in a lower nitrogen use efficiency (NUE)^7^.

NUE is an important indicator and is generally used to evaluate the fate of N in improving crop yields^8,9^. However, the unreasonable application of nitrogen fertilizer significantly lowers the nitrogen utilization rate. In China this rate is maintained at a level of 25%, which is significantly lower than the international level^10^. At the same time, the long-term use of chemical fertilizers not only leads to erratic yields^11^, but also triggers a decrease in soil pH value and organic matter, and causes instability of soil nutrients, leading to serious environmental degradation such as acid rain, groundwater pollution, soil acidification, and greenhouse gas emissions^6,12^.

Generally, both the form and the source of nitrogen fertilizers affect grain yield by regulating nitrogen transformations, changing nitrogen loss patterns and influencing NUE^13^. Application of organic fertilizers not only increases soil N content, but also enhances the storage of soil organic carbon and influences the pH and soil bulk density^8,12,14^. Moreover, long-term application of organic fertilizers simultaneously improved soil quality and fertility, and provides a solid foundation for promoting soil sustainable productivity^5,15,16^. Compared with the chemical fertilizer application, the combined application of manure and fertilizer increased the crop yield during the whole test period and raised the SOC and TN reserves^17,18^. Therefore, the combined application of organic and inorganic fertilizers has been commonly recognized^19^.

SOM plays an important regulatory role in the complex buffering processes in soils. Organic fertilizer application not only improves the soil physical characteristics^20^, but also promotes microbial activity and speeds up the degradation of SOM in soils^21^. To understand exactly the effect of organic fertilizer on soil fertility and NUE, location fertilization has been used to explain these changes^8^. But most researches with this aim were still focused on a one-year trial, and confirmed that SOM can be changed markedly depending on changes in weather and demonstrate a wide annual variability^22^. Thus, understanding the regulatory role of the application of organic fertilizer in soils with long-term location fertilization requires a series of assessments in nitrogen use efficiency.

Northeast Plain is one of the major grain production regions in China. However, long-term traditional cultivation and unreasonable use of fertilizers in the past 30 years have resulted in the degradation of soil quality, a decrease in pH and lower NUE. To overcome these obstacles, the application of organic fertilizer is an important choice. Although previous reports by long-term fertilization experiments in China have confirmed that the enhancement in soil fertility and yield were related to organic manure, we still lack detailed understanding of long-term organic fertilizer application on nitrogen effects in the brown soils of Northeast. According to 40 years’ data in this experimental site, the yield of the next 80 years was estimated by the Decision Support System for Agrotechnology Transfer-Cropping System Model (DSSAT-CSM), and it was concluded that the combined application of organic fertilizer and chemical fertilizer could effectively increase crop yield and reduce the yield reduction caused by climate^23^. Based on a long-term fertilization experiment (40 years) in Northeast Plain in China, a 2-year-location field experiment was performed by using four kinds of soils with ^15^N-labeled urea to investigate the effect of different long-term fertilizations on soil fertility, pH value and crop yields, as well as quantify the contribution of N fertilizer to current crop and the availability use of residual N in next-year crop growth in soils of different fertility. We expect that this study will provide a scientific guidance for reasonable application of organic-chemical fertilizers in soils which have had a long-term application of chemical fertilizers.

## Materials and methods

### Field site description

A long-term field location experiment was performed in 1979 at the Experimental Station of Shenyang Agriculture University, Liaoning Province in China (40º48′N, 123°33′E). The experimental site belongs to a region accompanying temperate, semi-humid climate with a mean annual temperature of 7.0–8.1 °C, and has a mean annual precipitation of 574–684 mm with the potential evaporation of 1,435.6 mm per year. The soil type is a typical brown earth loam containing 48% sand, 29% silt and 23% clay in the depth of 0–20 cm . Soil properties of 0–20 cm depth in 1979 were presented as follows: 6.5 pH in water (1:10), 15.9 g kg^−1^ organic matter, 0.8 g kg^−1^ total N, 0.38 g kg^−1^ total P, 21.1 g kg^−1^ total K, 6.5 mg kg^−1^ Olsen-P, 97.9 mg kg^−1^ available K, and 1.18 g cm^−3^ bulk density^23^.

### Experiment design

A total of 18 treatments in the experimental field site with micro-plots (length × width × height = 2 m × 1 m × 1 m) were established in 1979, laid out in randomized block desing with three replications per treatment. The soils representing the five fertility levels from the trial were selected, with a total of fifteen micro-plots. In brief, these soils include the treatments of unfertilized (CK), inorganic N fertilizer (N), inorganic N, P, and K fertilizer (NPK), manure plus inorganic N, P and K fertilizer (M_1_NPK), and two folds amounts of manure and inorganic N, P and K fertilizer (M_2_NPK). The crop rotation model is soybean-maize-maize by one crop per year, with sowing times in early May and harvesting in early October. Before soybean sowing each year the N, P, and K fertilizers were applied at rates of 30 kg N ha^−1^, 90 kg P_2_O_5_ ha^−1^, and 90 kg K_2_O ha^−1^. Before maize sowing each year the N, P and K fertilizers were applied at rates of 120 kg N ha^−1^, 60 kg P_2_O_5_ ha^−1^, 60 kg K_2_O ha^−1^. Manure fertilizer (pig manure contained total C, N, K and P of 83.5 g kg^−1^, 7.2 g kg^−1^, 8.6 g kg^−1^ and 10 g kg^−1^, respectively, based on the dry matter) was just applied at rates of 27 t ha^−1^ and 54 t ha^−1^ in M_1_NPK and M_2_NPK treatment before planting maize each year, respectively. The urea was applied as N fertilizer, the superphosphate was applied as P fertilizer, and the potassium sulfate was applied as K fertilizer.

To eliminate the statistic errors from environment factors in the field trials, twelve Poly-vinyl-chloride (PVC) material containers (length × width × height = 0.9 m × 0.37 m × 0.6 m) were vertically inserted into each micro plot representing the treatments of four fertility levels to perform the experiment on April 29, 2016. A height of 3 cm in each container was spaced apart from the soil surface to prevent soil and runoff from entering the containers. Before sowing soybean, the topsoil (1–20 cm depth) in each container was completely mixed with urea of ^15^N-labeled by 20.2% of atom at the rate of 30 kg ha^−1^ (+ N treatment), and the P and K fertilizers were also mixed these topsoil from each micro-plot at the rates described above, then the fertilized soils were returned to the containers, and finally soybean seeds (Liaodou 15) of 4 grains were sown in each container, while a total of 20 soybean seeds were sown outside the container. The soybean grains were harvested in early October 2017. Two grains of maize seeds (Dongdan 6531) were planted in each container in early May 2018, and a total of 10 maize seeds were planted outside the container. The application rates of N, P, K and manure both inside and outside the containers were identical.

### Plant and soil sampling

Four soybean plants were removed from each container with a shovel, and collected by the forms of stem and leaf, root, grain, and shell. These soybean tissues were cleaned by water and dried in oven at 60 ºC, when a constant weight was used to determine the dry-matter biomass. These plant samples of each part were smashed and sieved through a 100 mesh to determine total N contents and ^15^N abundance after digestion. The remaining soybean plants in the micro plot were used to measure yield and dry matter accumulation. The maize plants were collected by the form of root, stem, leaf, cob, and grain, and further treated by the same way as the soybean plants.

Three soil cores (2 cm diameter) were placed in increments of 20 cm and mixed well into a single soil sample from 0 to 80 cm depth of each container after harvesting soybean and maize. Soil samples were air-dried naturally at room temperature, and both visible roots and gravels were removed. The samples were sieved through a 100 mesh to determine total N contents, ^15^N abundance, P, K, soil organic matter and pH valve with standard measurement method.

### Soil and plant sample analysis

The measurements of total N and the ^15^N abundance in plant and soil samples were determined by EA-IRMS (Elementar vario PYRO cube coupled to IsoPrime 100 Isotope Ratio Mass Spectromenter, Germany). Available P in soils were extracted with 0.5 mol L^−1^ NaHCO_3_ and determined by using the molybdenum blue method^24^. Soil pH was measured with deionized water (1:5 soil/water)^17^. SOM was determined using Walkely Black method^25^.

### Calculations

The percentages of N derived from fertilizer N (Ndff) were calculated by the following formula:

1 $$\mathrm{Ndff}\left({\%}\right)=\frac{{at\%}^{15}N\, excess \,in \,plant \,or \,soil}{{at\%}^{15}N \,excess \,in \,fertilizer}\times 100$$

Both accumulation of N and recovery in plants were calculated by the following formula:

2 $${\mathrm{N}}_{\mathrm{uptake}}\left({\mathrm{kg \,hm}}^{-2}\right)=\frac{{\mathrm{W}}_{\mathrm{plant}}\times {\mathrm{N}}_{\mathrm{plant}}}{1000}$$

3 $${\mathrm{N}}_{\mathrm{plant}-\mathrm{fertilizer}} ({\mathrm{kg\, hm}}^{-2})={\mathrm{N}}_{\mathrm{uptake}}\times {\mathrm{Ndff}}_{\mathrm{plant}}$$

where W_plant_ (kg hm^−2^) represents the weight of plant dry matter of grain, stem, leaf, root, pod, stalk, and whole plant; N_plant_ (g kg^−1^) represents the content of total N of corresponding plant, and N_plant-fertilizer_ represents plant N from fertilizer.

The amounts of residue N in soil N_soil-residue_ (kg hm^−2^) were calculated by the following formula:

4 $$\mathrm{c}\left({\mathrm{kg \,hm}}^{-2}\right)={\mathrm{W}}_{\mathrm{soil}}\times {\mathrm{N}}_{\mathrm{soil}}\times {\mathrm{Ndff}}_{\mathrm{soil}}$$

where W_soil_ (kg hm^−2^) represents the weight of soil (20 cm profile) per hectare, and N_soil_ (g kg^−1^) represents the content of total N of soil.

The recovery rates of fertilizer N in the plant (R_plant_) were calculated by the following formula:

5 $${\mathrm{R}}_{\mathrm{plant}}\left({\%}\right)=\frac{{\mathrm{N}}_{\mathrm{plant-fertilizer}}}{{\mathrm{N}}_{\mathrm{input}}}\times 100{\%}$$

where N_input_ (kg hm^−2^) represents the total applied amounts of N before soybean sowing.

The recovery rates of fertilizer N in the soil (R_soil_) were calculated by the following formula:

6 $${\mathrm{R}}_{\mathrm{soil}}\left({\%}\right)=\frac{{\mathrm{N}}_{\mathrm{soil-residue}}}{{\mathrm{N}}_{\mathrm{input}}}\times 100{\%}$$

The loss rates of N in the crop-soil system (R_loss_) were calculated by the following formula:

7 $${\mathrm{R}}_{\mathrm{loss}}\left({\%}\right)=1-{\mathrm{R}}_{\mathrm{plant}}-{\mathrm{R}}_{\mathrm{soil}}$$

### Statistical analysis

All data in both the figures and tables were presented by the means of three replicates with standard deviation. A One-way ANOVA was used to test the effects of fertilizer and residue fertility on contribution of crops after a long-term fertilization. All data were analyzed with SPSS 19.0 and the graphics were drawn through a software of SigmaPlot 12.

One-way ANOVA was used to analyze statistically the differences in total N, ^15^N recovery rate in crops and in soils, and the changes in crop biomass and yields after the long-term fertilization treatment or manure fertilizer application. The least significant difference (LSD) tests were used to assess differences at a level of *P* < 0.05.

## Results

### Higher input of manure significantly increases crop yield and N uptake

**Figure 1:**
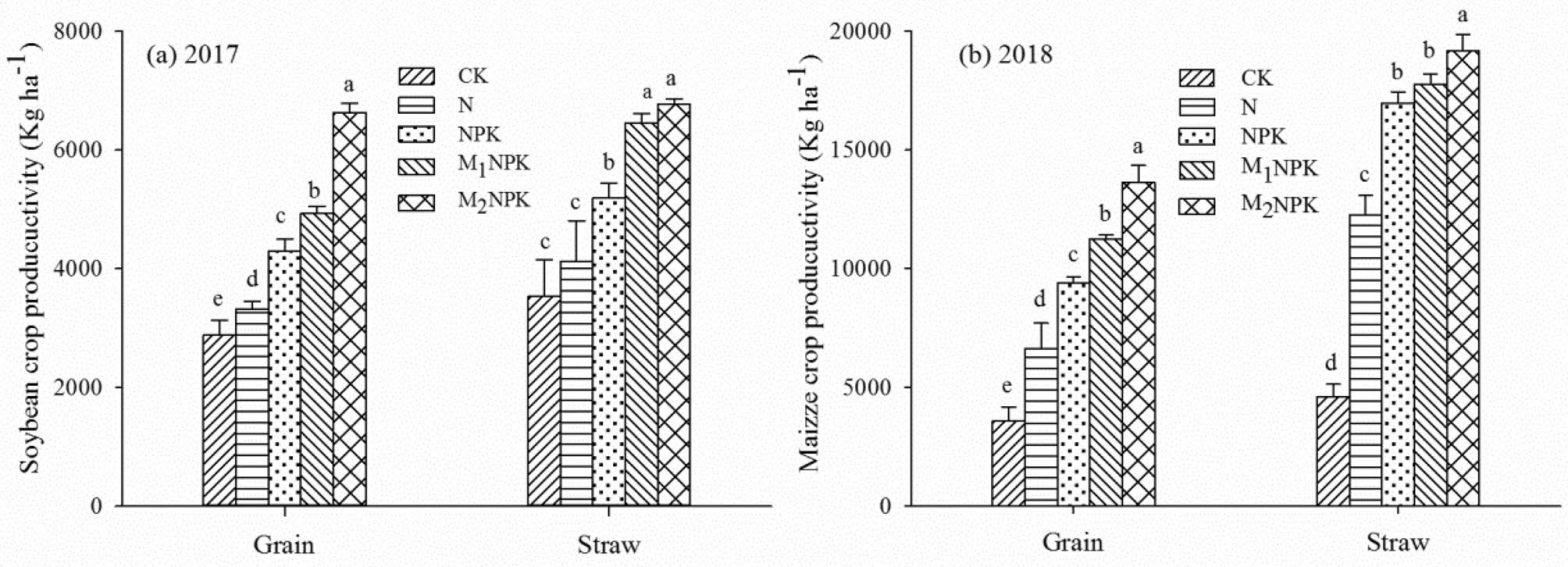
Mean yields of soybean (**a**) and maize (**b**) in different fertilization treatments in 2017 and 2018. The same letters indicate an insignificance between the fertilization treatments at *P* < 0.05. Error bars show standard deviations of means (n = 3).

Different fertilization treatments from the location experiment sites of 40 years demonstrated significant differences in crop yields. Both the M_1_NPK and M_2_NPK treatments revealed remarkably higher yields of grains and straw than the CK, N and NPK treatments in 2017 (*P* < 0.05), and also demonstrated a similar pattern in 2018 (Fig. 1). Data showed that the maize grain yields were commonly higher than soybean for all treatments, and the M_2_NPK treatment generated the highest grain yield because of better interaction between higher organic fertilizer and balanced chemical fertilizer. Maize yields in the treatment with N application increased by 84.6% compared with CK, but the soybean yields in the same treatment were only increased by 15.5%.

In the case of N uptake, the M_2_NPK treatment showed the highest accumulation at the harvesting stage of soybean and maize. The amounts of N uptake were 668.5 kg ha^−1^ in soybean and 594.8 kg ha^−1^ in maize (Table 1). Different treatments showed a significant difference in soybean N uptake; the amounts of N uptake in the M_1_NPK and M_2_NPK treatments were higher than those in the N and NPK treatments, although no significant differences between the M_1_NPK and M_2_NPK treatments were observed. Total plant N in soybean grain are ranged from 66.3 to 72.1%, while the amounts of N uptake in maize only account for 39–50.9% (Table 1).

**Table 1:** N uptake in soybean and maize in 2 years.

Treatments	N uptake (kg ha^−1^)									
	2017					2018				
	Grain	Stem + Leaf	Pod	Root	Total	Grain	Stem + Leaf	Stalk	Root	Total
CK	201.6 ± 17.9^e^	34.3 ± 5.5^c^	24.8 ± 4.6^c^	16.8 ± 0.7^c^	305.5 ± 24^e^	85.7 ± 7.6^d^	55.1 ± 6.3^c^	25.4 ± 4.3^c^	18.7 ± 3.1^d^	184.9 ± 6.3^c^
N	234.3 ± 8.6^d^	37.7 ± 6.7^c^	33.8 ± 4.9^b^	19.0 ± 1.9^c^	324.8 ± 6.2^d^	168.3 ± 8.4^c^	101.9 ± 16.4^b^	37.1 ± 0.2^b^	26.6 ± 0.8^c^	333.9 ± 20.1^b^
NPK	303.3 ± 21.9^c^	64.7 ± 7.8^b^	40.8 ± 1.9^b^	32.4 ± 4.4^b^	441.2 ± 29.9^c^	154.7 ± 16.6^c^	106.3 ± 30.4^b^	52.6 ± 14.7^b^	31.3 ± 5.4^c^	344.8 ± 59.0^b^
M_1_NPK	366.7 ± 8.6^b^	92.8 ± 10.8^a^	59.8 ± 6.5^a^	33.8 ± 2.3^b^	553.1 ± 8.5^b^	211.0 ± 31.8^b^	196.1 ± 78.8^a^	74.8 ± 16.5^a^	68.3 ± 25.4^b^	550.3 ± 147.2^a^
M_2_NPK	474.0 ± 16.5^a^	82.5 ± 8.7^a^	66.0 ± 4.6^a^	50.0 ± 5.6^a^	668.5 ± 15.9^a^	231.7 ± 43.9^a^	210.7 ± 81.8^a^	70.5 ± 17.1^a^	82.0 ± 23.4^a^	594.8 ± 163.2^a^

*Note* Means followed by the same letter within the same column represent no significance (*P* < 0.05).

### Application of manure improves the availability of nutrients in soils

The soil pH, SOM, total N, Olsen P, and available K in the soil layer of 0–20 cm depth are summarized in Table 2. The pH values are ranged from 5.11 to 6.28, which were lower than the initial value in 1979, and the N treatment revealed the lowest pH value. Manure application combining with chemical fertilizers increased the pH value compared to that of the application of chemical fertilizers (Table 2). The amounts of SOM, total N, Olsen P and available K in the treatments of chemical fertilizer combined with organic fertilizer were significantly higher than those in the treatments of chemical fertilizers, with the M_2_NPK treatment demonstrating the highest accumulation of SOM, available N, P and K. However, the N and NPK treatments did not show significant differences in the contents of SOM, total N, and available K (Table 2).

**Table 2:** Chemical properties of the five soils in 1979, 2017 and 2018.

Years	Treatments	pH	Organic matter (g kg^−1^)	Total N (g kg^−1^)	Olsen P (mg kg^−1^)	Available K (mg kg^−1^)
1979		6.5	15.9	0.8	6.5	97.9
2017	CK	5.87 ± 0.02^c^	13.52 ± 0.11^d^	0.93 ± 0.03^d^	2.1 ± 0.20^d^	83.66 ± 4.33^c^
	N	5.11 ± 0.04^e^	15.10 ± 0.09^c^	1.05 ± 0.04^c^	2.0 ± 0.26^d^	91.74 ± 6.52^c^
	NPK	5.44 ± 0.04^d^	15.41 ± 0.84^c^	1.10 ± 0.08^c^	21.2 ± 6.73^c^	95.34 ± 2.27^bc^
	M_1_NPK	5.93 ± 0.01^b^	20.14 ± 0.61^b^	1.51 ± 0.02^b^	92.7 ± 2.4^b^	124.28 ± 10.96^ab^
	M_2_NPK	6.27 ± 0.03^a^	23.14 ± 0.86^a^	2.04 ± 0.003^a^	146.3 ± 2.35^a^	140.68 ± 4.14^a^
2018	CK	5.94 ± 0.03^c^	13.48 ± 0.42^d^	1.01 ± 0.03^d^	1.7 ± 0.17^d^	77.13 ± 3.77^c^
	N	5.20 ± 0.03^e^	15.12 ± 0.44^c^	1.11 ± 0.03^c^	1.9 ± 0.20^d^	90.99 ± 3.20^b^
	NPK	5.41 ± 0.03^d^	15.53 ± 0.87^c^	1.25 ± 0.03^c^	18.8 ± 0.87^c^	107.44 ± 7.40^b^
	M_1_NPK	6.04 ± 0.04^b^	20.41 ± 0.71^b^	1.60 ± 0.03^b^	83.6 ± 1.65^b^	141.89 ± 10.36^a^
	M_2_NPK	6.28 ± 0.04^a^	22.95 ± 0.64^a^	2.21 ± 0.03^a^	120.9 ± 6.70^a^	133.19 ± 2.57^a^

*Note* Means followed by the same letter within the same column indicate no significance (*P* < 0.05).

### Manure promotes the ^15^N recovery both in crops and soils

As shown in Table 3, Nitrogen use efficiency was significantly affected by the application of organic manure. Nitrogen use efficiency in soybean ranged from 18.3 to 32.5%. Compared with N, the NUE in soybean were increased by 31.4, 45.3 and 77.4%, respectively. Manure application significantly lowered the N loss rates. The highest loss rate in the N treatment was 22.3%, and the lowest loss rate in M_2_NPK treatment was 5.5%, indicating that the combination application of organic fertilizer and chemical fertilizers could significantly promote the utilization rate of nitrogen fertilizers and reduce the loss of nitrogen fertilizers.

**Table 3:** The fate of ^15^N fertilizer in soybean at harvesting stage.

Treatments	Crop uptake		Soil residual		Loss	
	kg ^15^N ha^−1^	%	kg ^15^N ha^−1^	%	kg ^15^N ha^−1^	%
N	5.50^c^	18.3^c^	17.82^b^	59.4^b^	6.68^a^	22.3^a^
NPK	7.22^b^	24.1^b^	19.17^a^	63.9^a^	3.61^b^	12.0^b^
M_1_NPK	8.00^b^	26.6^b^	18.97^a^	63.2^a^	3.04^b^	10.1^b^
M_2_NPK	9.75^a^	32.5^a^	18.61^a^	62.0^a^	1.64^c^	5.5^c^

*Note* Means followed by the same letter within the same column represent no significance (*P* < 0.05).

**Figure 2:**
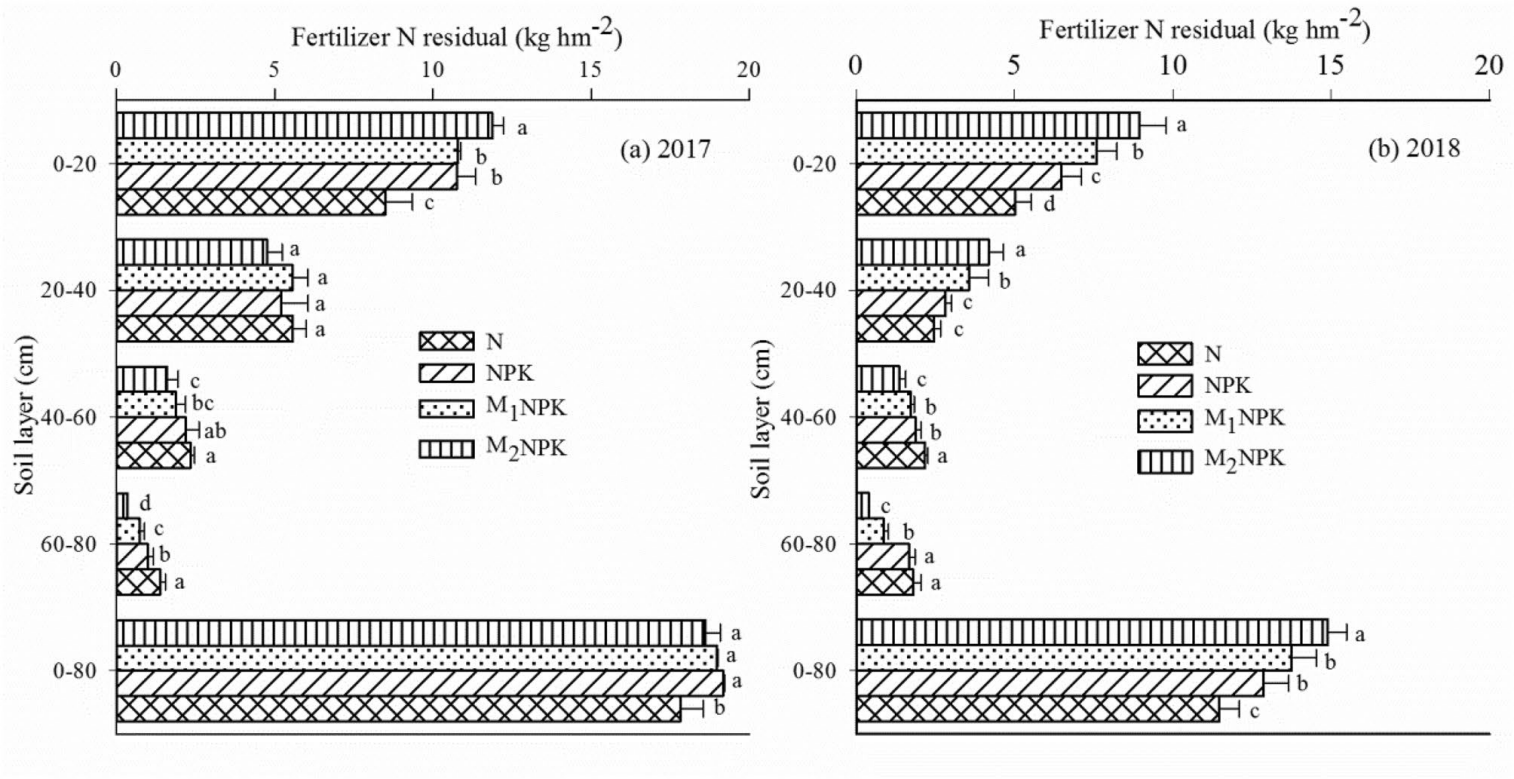
The distributions of residual ^15^N from N fertilizer application at the soybean harvesting stage in 2017 and at the maize harvesting stage in 2018. Values are the means of the three replicates of each treatment. Vertical bars represent standard errors.

^15^N measurement showed that the 59.4–63.9% of applied ^15^N fertilizer were remained in soil at the soybean harvesting stage. The recovery of ^15^N in the 0–20 cm soil layer was significantly higher than those in the 20–40 cm and 40–60 cm layers, and nearly half of ^15^N remained as residuals in the 0–20 cm soil layer, the same pattern was found at the maize harvesting stage (Fig. 2).

### Manure confers an enhancement in nitrogen use efficiency

Measurement showed that the ^15^N recovery rate of N fertilizer in soybean was 18.3–32.5% in the first growth season, which demonstrates a remarkable difference between the treatments. In the case of the same amounts of N fertilizer application, the recovery rate of ^15^N was expressed by an order of M_2_ > M_1_ > M_0_. In the case of the second year, the recovery rate of residual-derived N in maize displayed a decrease of 6.1–8.8%, and the highest recovery rate of residue-derived N was the M_1_NPK, but no significant difference was observed compared with the other treatments.

**Figure 3:**
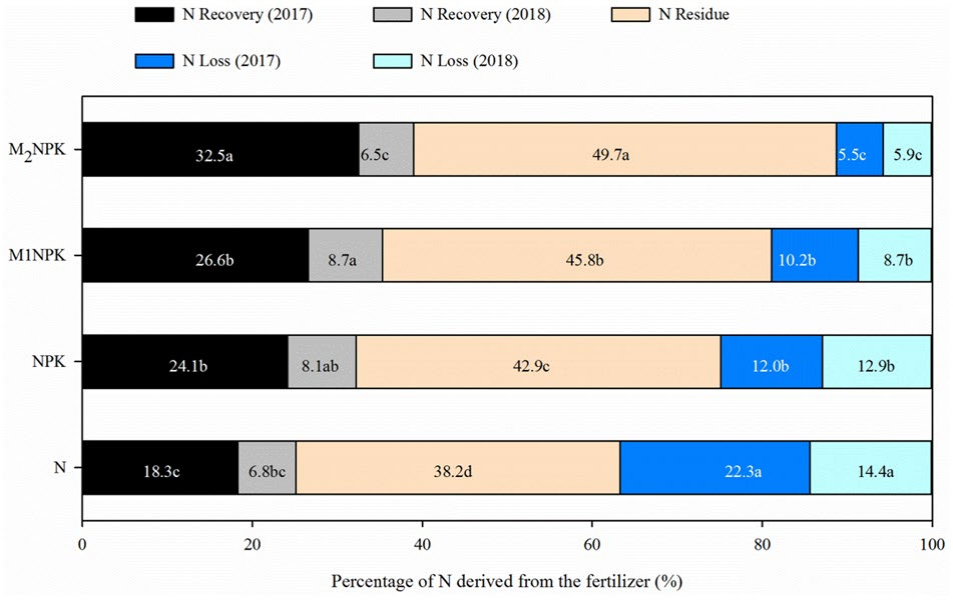
The fate of ^15^N-labelled urea in two crop seasons. Values are the means of the three replicates of each treatment. Vertical bars represent standard error.

The recovery of residual nitrogen in the soil dominated the main proportion of total nitrogen recovery in each treatment, as 59.4–63.9% of the residual nitrogen still existed in the soil as a potential N source after one year, and this range was decreased to 38.2–49.7% after two years (Fig. 3). The combined application of organic fertilizer and chemical fertilizers can significantly increase the amounts of residual N in soils compared with the application of only chemical fertilizer. Meanwhile, the rates of loss N in the treatments ranged from 5.5 to 22.3% in the first year and 5.9–14.4% in the second years, and revealed significant differences. Data also showed that manure application effectively lowered the rate of loss N.

## Discussion

### Crop yield, soil properties and N recovery

Increased N uptake and crop yield is generated by fertilizer application^26–28^. Differences in grain yield and N uptake between the five treatments indicate significantly different effects on soil fertility and crop yield after long-term different fertilization. The present study clearly demonstrated that manure application revealed a great beneficial income in both the yield and N uptake in the soybean-maize rotation system. M_1_NPK and M_2_NPK treatment increased soybean productivity by 12.8–32.5% and 35.2–49.8% respectively compared to chemical fertilizer, and the maize yield increased by 16.6–50.0% and 31.2–51.3%, respectively (Fig. 1). Which revealed a similar result to the report from Afreh et al.^8^. Organic fertilizer combined with chemical fertilizer significantly increased crop yield^29^, that might not only be ascribed to the additional application of organic fertilizer increased nutrients , but also the increase of soil organic matter and the increase of soil nutrient availability^30^. Similar conclusions have been reported previously^5,15^. The soybean yield difference between the CK and N treatments was lower than maize yield, indicating that the rotation with soybean can effectively maintain crops yield because legumes plant soybean could effectively fix nitrogen by symbiotic nitrogen fixation system, thus reducing the requirement for N fertilizer^31^.

Compared to chemical fertilizer, chemical fertilizer combined with organic fertilizer significantly increased the amounts of SOM, total N, Olsen P and available K (Table 2). Long-term organic fertilizer application has been confirmed to play a positive regulatory role in maintaining nutrient balance and soil physical properties^16^. Our study shows that the soil pH value in the NPK treatment was decreased from 6.5 to 5.29, while the M_2_NPK still reached a pH of 6.27 after long-term fertilization of 40 years, as reported previously Xie et al.^32^ and Chen et al.^33^. Shi et al.^14^ also found that application of manure could increase pH buffering capacity and alleviate soil acidification. Generally, the N uptake in maize has a closely associated with the aboveground biomass. Therefore, the manure addition promoted the N uptake and provided better sustainability of soil fertility and crop yield than the inorganic fertilizer did^34^. Similar results were also observed through a 21-year soybean-wheat cropping system in the Indian mid-Himalayas^35^.

**Figure 4:**
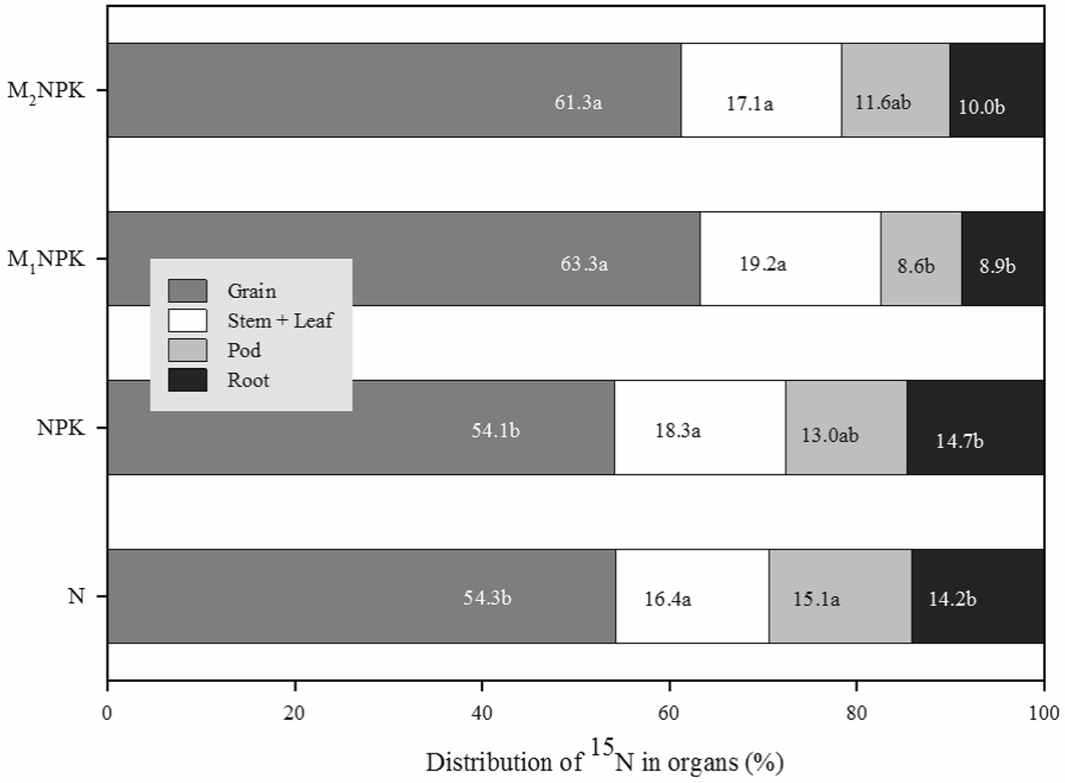
Effects of N application on the distribution of ^15^N in soybean organs. The different letters on bars represent significant differences at a level of 5%.

In the first season, plant N derived from fertilizer were ranged from 18.3 to 32.5% after applying ^15^N labeled fertilizer. Plenty of research reported that ^15^N recoveries ranging from 20 to 63% in the crop are related to the crop rotation, fertilization timing, irrigation, and rainfall conditions^4,36–38^. The ^15^N tracer technique clearly showed that the application of chemical fertilizer with manure revealed a higher recovery of N fertilizer in crops (Table 3) and lowered percent potential losses of N fertilizer. Previous studies have reported that a total of 66.9–69% of the absorbed N^15^ in crops were accumulated in grains under different fertilization treatments^28^. In our study, the amounts of the ^15^N absorbed in crop grains were ranged from 54.1 to 63.3%, and organic fertilizer treatments were significantly higher than chemical fertilizer treatments, suggesting that two treatments with manure increased the proportion of the ^15^N uptake in grains (Fig. 4).

Our data showed that although soybean absorbed a part of fertilizer N, most of ^15^N fertilizer still remained in the soil, indicating that the accumulated N in soybean mostly came from the intrinsic N in soil and symbiotic nitrogen fixation system. Wang et al.^28^ reported that a total of 48.3–51.3% of residual ^15^N was still remaining in the soil layer of 0–200 cm at the stage of harvesting, and approximately half of residual N was moved into the soil layer of 0–20 cm, which was the similar to our results. In this study, using ^15^N tracer technique confirmed the effect of fertilizer application on residual amounts of fertilizer N and the downward movement of N fertilizer in soils. In our study, a total of 59.4% and 63.9% of fertilizer ^15^N remained in the layer of 0–80 cm depth in the N and NPK treatments respectively at the stage of harvesting soybean, and the proportion of residue ^15^N was increased with the extension of the soil layer depth compared with the residuals of 63.2% in the M_1_NPK treatment and 62.0% of the fertilizer ^15^N in the M_2_NPK treatment, the same pattern was found at the maize harvesting stage (Fig. 2), indicating that manure application effectively lowers the down movement of the N fertilizer in soil. This observation shows a similar result to previous report^39,40^, who thought that this translocation is most likely related to the N immobilization in the soil organic matter.

Many reports confirmed that while most of the fertilizer N remained in the soil after the first season, only a small amount of residue N was absorbed by the second crop^26,41,42^. In our report, only total of 6.5–8.7% of the initial applied N was utilized by maize as shown in Fig. 3. Generally, N fertilizer is more readily available to crops compared to the residual N, meanwhile there was not much difference in ^15^N recovery from residual N in plants in several soils^42^. Our study also confirmed this conclusion by investigating four treatments with different fertility levels. Sebilo et al.^4^ also found that the fate of ^15^N fertilizer that a total of 61–65% of the applied fertilizer N were taken up by plants under a long-term sugar beet-winter wheat rotation system, and succeeding crop utilized nearly 15% of residual N in the fertilization soil of 29 years although the residual fertilizer N recovery in the follow-up crops is relatively low compared with that in the first season crops. The reason might be that fertilizer N is more easily absorbed by the crops, thus reducing the loss pathway of N naturally in soil^41,42^. Commonly, residual fertilizer N is composed of various nitrogen forms, and the majority of residual fertilizer N existed in the organic form after the first crop harvesting^43^, and is transformed into more stable soil pools. As previously reported, a total of 12–15% ^15^N of the fertilizer N was still remaining in the soil organic matter after the 30-year application of ^15^N tracing in soils^4^.

### The mechanisms for increased crop yield and NUE under organic fertilizer

The use of chemical fertilizer combined with organic fertilizer on yield has been widely studied^14,35^. It is well-known that organic fertilizer can improve the soil’s physical and chemical properties^44^, increase soil nutrient availability and promote the crop growth. In our study, organic fertilizer significantly increased SOM by 23.5–34.8% compared to chemical fertilizer and results of increasing soil organic matter based on organic fertilizers are also supported by many other long-term fertilization experiments^45,46^. The manure contained alkaline substances that neutralized soil acidity, which led to an increase in soil pH^14^. Organic materials from manure transformed into soil organic matter through a series of complex biochemical reactions after the manure was applied to the soils. The soil organic matter contained many organic functional groups, such as carboxylic and phenolic groups^47^. The dissociation of these acidic functional groups increased the soil negative charge and thus soil CEC, and also increased the resistance to acidification. On the one hand, the application of organic fertilizer stabilized the soil structure, slowed down the N migration, and effectively reduced the fertilizer N loss; on the other hand, the improvement of soil microbial biomass and activity was conducive to the immobilization of N in the pre-growing stage and the gradual remineralization afterwards^48^. Therefore, the application of organic fertilizer was beneficial to improve the NUE, which also increased the crop yield.

At present, Chinese agriculture mainly depends on high-input chemical N fertilizer rather than organic fertilizer, which results in reduced NUE and soil quality deterioration^38,49^. In order to ensure food demand and save resources, it is necessary to recycle the abundant organic resources and reduce the amount of fertilizer. The combination of organic fertilizer and nitrogen fertilizer is in line with the government's goal of ensuring food security and reducing environmental hazards, which is economically attractive to farmers. In China, there is a huge amount of pig manure, which is easy and cheap for farmers to obtain. Therefore, the application of organic fertilizers is an effective and promising approach for developing sustainable agricultural.

## Conclusions

The results from the 40-years long-term location experiment indicated that the combined application of chemical and organic fertilizer is an effective fertilization practice in order to sustain high crop yield, increase SOM, total N, Olsen-P, and available K, and alleviate soil acidification. By using the ^15^N tracing technique, the organic fertilizer can significantly improve the NUE in the first crop season and decrease potential N loss. However, there were no significant differences in the recovery of residual ^15^N in the second season. It is worth noting that as a potential N pool, the large amount of ^15^N remaining in the soil after two crops can play an important role in contributing to future crops. Our findings suggest that the application of organic fertilizer can be an effective and promising practice for developing sustainable agriculture in order to produce a higher yield and more effectively use resource, and farmers could adopt the rational combination of organic fertilizer with appropriate chemical fertilizer.
